# Testing Accommodation: The Case for Nott Dynamic Retinoscopy in Orthoptic Assessments

**DOI:** 10.22599/bioj.550

**Published:** 2026-04-28

**Authors:** Sonia Toor, Jessica E. Francis

**Affiliations:** 1 School of Allied Health Professions, Pharmacy, Nursing and Midwifery, University of Sheffield, United Kingdom; 2 Sheffield Teaching Hospitals, United Kingdom

**Keywords:** accommodation, Plusoptix, dynamic retinoscopy, RAF Rule

## Abstract

**Introduction:**

Accommodation is most commonly assessed using the RAF Rule. However, this subjective test has multiple sources of error, and an objective measurement of accommodation using Nott dynamic retinoscopy may be a more appropriate test to use. This study aimed to compare Nott dynamic retinoscopy with the RAF Rule and the Plusoptix PowerRef III (gold standard) after a period of close work, to determine whether this is a more suitable test for measuring accommodation.

**Methods:**

Accommodation was measured in 25 students before and after 30 minutes of close work using the RAF Rule, Nott dynamic retinoscopy, and the Plusoptix. The RAF Rule measured the amplitude of accommodation when viewing N5 print. Nott dynamic retinoscopy assessed the lead/lag of accommodation using the UC Cube with N5 print at 40 cm. The Plusoptix measured objective accommodation (slope and response) in binocular and uniocular conditions.

**Results:**

The Plusoptix and Nott dynamic retinoscopy detected accommodative lag within normal limits (under 1D), with no significant change after close work (p > 0.05). No significant change in accommodation after close work was found with the RAF Rule tested uniocularly (p > 0.05); however, a significant decrease in amplitude of accommodation from 12.85D to 11.26D was found when tested binocularly. The Plusoptix confirmed that this was not due to convergence fatigue (p > 0.05).

**Discussion:**

The measurement variations observed with the binocular RAF Rule confirm that the subjective nature of the RAF Rule test makes it more prone to errors. Nott dynamic retinoscopy is comparable to the gold standard Plusoptix; therefore, we suggest that Nott dynamic retinoscopy should be used in the orthoptic assessment of accommodation.

## Introduction

Accommodation is most commonly assessed in orthoptic clinics as the near point of accommodation (NPA) using the push-up method on the RAF Rule. This method is simple to use and provides a quick measurement. However, a literature review by Burns *et al*. (2020) highlighted that the NPA can be affected by factors such as examiner bias, reaction time and instrument error. They listed 15 instrument errors, including ambiguity about which part of the sliding component indicates the reading, variability in the luminance of the text, effect of differing facial anatomy, and positioning of the RAF Rule. While the NPA provides information about the maximum accommodative ability, it does not reflect accommodative behaviour during sustained, real-life visual tasks, such as prolonged close work, which is more relevant to patient symptoms. The RAF Rule is also a subjective assessment of accommodation and, therefore, is prone to patient error.

An objective measurement, using equipment such as the Shin-Nippon or Grand Seiko autorefractor, is required to assess accommodation accurately. The subjective push-up method and a modified version of this have been found to overestimate the amplitude of accommodation (converted from the NPA) compared with the Grand Seiko (Anderson and Stuebing, 2014; Chen et al., 2019). Anderson *et al*. (2024) found that the subjective push-up method is not an accurate assessment of accommodation, diagnosing accommodative insufficiency when normal levels of accommodation were detected using the Grand Seiko, which suggests it is a poor diagnostic tool. Nevertheless, this method continues to be used. The drawback of such equipment, however, is that it is large and expensive, and therefore not readily available in clinics.

Dynamic retinoscopy is also an objective test of accommodation that may be a more appropriate clinical test, but it is often overlooked (Hunter, 2001; Bhalla and Mohan, 2014). This test uses a retinoscope, which is inexpensive and already available in all clinics, to assess the retinoscopic reflex and determine the level of accommodation. Measures of accommodative lag or lead provide information about the accuracy of the accommodative response during near tasks, at functional working distances rather than at threshold levels. The two most used methods of dynamic retinoscopy are Nott dynamic retinoscopy and the Monocular Estimate Method (MEM). Nott dynamic retinoscopy provides valid and repeatable results (McClelland and Saunders, 2003) and is quicker and simpler to perform than MEM dynamic retinoscopy. In Nott dynamic retinoscopy, neutralisation is achieved by adjusting the working distance of the retinoscope, whereas MEM dynamic retinoscopy requires introducing lenses of varying strengths before the eye to achieve neutralisation. This increases procedural steps and may disrupt the accommodative response, making Nott dynamic retinoscopy better suited to routine clinical use.

Nott dynamic retinoscopy is more accurate than the subjective push-up method, as the latter overestimates accommodative ability (León et al., 2024). However, results have been more variable when comparing Nott dynamic retinoscopy with an autorefractor. McClelland and Saunders (2003) found Nott dynamic retinoscopy to be repeatable and comparable with the Shin Nippon autorefractor, although the Correction of Myopia Evaluation Trial 2 Study Group (2009), using the Grand Seiko, found poor agreement between the tests, with Nott dynamic retinoscopy underestimating the lag of accommodation. Although these autorefractors have their advantages—such as the Grand Seiko’s wide operating range—they take single measurements of accommodation, capturing the accommodative response at specific time points and only in one eye. The difference in findings between these studies could be due to these limitations. Using the Plusoptix PowerRef III photorefractor would be more advantageous, as it measures accommodation in both eyes and continuously at 50Hz, with the added benefit of simultaneously measuring convergence and pupil size.

Assessment of accommodation forms a vital part of patient diagnosis and care, yet research suggests that the RAF Rule is a poor diagnostic tool (Burns et al., 2020; Anderson and Stuebing, 2014; Anderson et al., 2024; León et al., 2024). Nott dynamic retinoscopy and objective autorefractors offer assessments that are potentially more clinically relevant than the NPA, though their comparability in assessing accommodation is unclear. Previous studies typically compare two methods of testing accommodation; however, to determine whether Nott dynamic retinoscopy is a more appropriate test, it should be compared with the RAF Rule and an objective test that can be considered the gold standard, such as the Plusoptix PowerRef III. We have a Plusoptix lab setup that also allows us to remove disparity cues, the main cue that drives accommodation (Horwood and Riddell, 2008), enabling a more detailed assessment of accommodation under binocular and uniocular conditions—an approach not undertaken in previous studies. Research comparing accommodation tests typically involves comparing measurements at a single time point or assessing the test-retest repeatability, under the assumption that there is no change in measurement upon repeat testing. The RAF Rule measures the NPA or the amplitude of accommodation, whereas Nott dynamic retinoscopy measures the lag of accommodation, making direct comparisons difficult. Instead, to determine the accuracy of the tests, they could be repeated after a period of close work. Therefore, this study aimed to compare Nott dynamic retinoscopy with the RAF Rule and Plusoptix PowerRef III, using a period of close work to elicit a possible change in accommodation, to determine whether this is a more suitable test for measuring accommodation.

## Methods

The study adhered to the Declaration of Helsinki and received ethical approval from the University of Sheffield Research Ethics Committee. Undergraduate orthoptic students were recruited to take part in the study using adverts on their Virtual Learning Environment. Informed consent was obtained from all participants.

The inclusion criteria were: aged 18–35 years to exclude early presbyopia; distance VA of ≤ 0.20 logMAR in each eye to ensure the vision was good enough to view the targets; no refractive error or must be wearing refractive correction (glasses or contact lenses) as uncorrected hypermetropes and myopes have different accommodative demands; no manifest deviation and stereoacuity of 300″ tested with the Frisby Stereotest to prove binocularity.

Accommodation was assessed by a qualified orthoptist using the Plusoptix PowerRef III, Nott dynamic retinoscopy, and the RAF Rule. The tests were counterbalanced. Thirty minutes of close work was performed before the tests were repeated in the same order. Participants undertook any close work of their choice, so the work included a mix of screen- and paper-based tasks. Participants were monitored throughout this period by the assessor.

### Plusoptix PowerRef III

**Figure 1:**
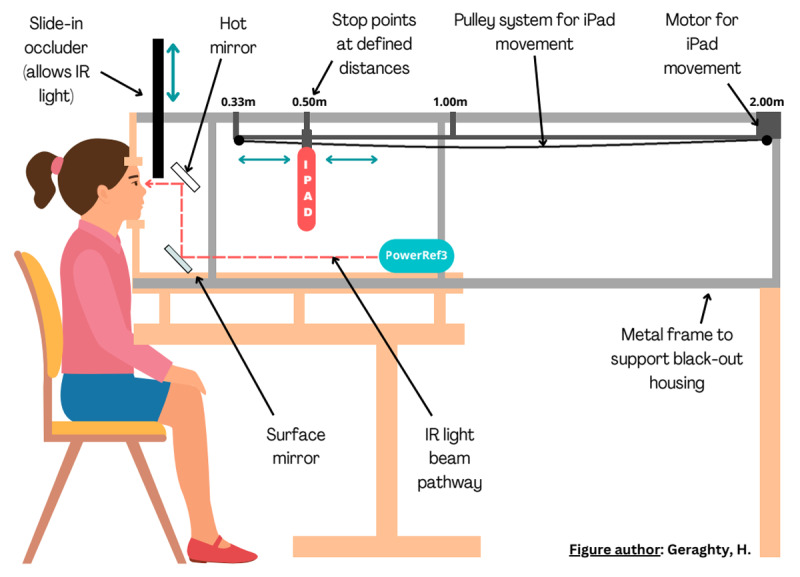
The Plusoptix PowerRef III lab setup.

The Plusoptix PowerRef III measures the refraction in both eyes (later converted to accommodation) at 50Hz (simultaneously measuring vergence and pupil size). Figure 1 illustrates the Plusoptix lab setup. The Plusoptix is placed within a black housing unit, below the participant’s line of sight, but uses a mirror system that reflects infrared light to allow refraction of the eyes. This setup prevents interference with the target pathway. The target was a single line of LogMAR letters, presented at eye level on an iPad suspended within the unit on a motorised beam at four distances set in a pseudo-random order (2 m, 50 cm, 1 m, 33 cm) representing demands of 0.5D, 2D, 1D and 3D. It is seen through a ‘hot mirror’ which is transparent to visible light but reflects infrared light, allowing the participant to see the target through the mirror while the Plustoptix captures refraction data.

The lab setup allows manipulation of disparity cues, which were eliminated when the participant was occluded using an infrared filter placed in front of the right eye. This obscured the participant’s view of the target in the same way as if an occluder was placed directly in front of that eye, but allowed infrared rays through so measurements could still be taken from both eyes. This allowed us to assess accommodation binocularly and uniocularly. The two conditions were counterbalanced. Participants were simply instructed to watch the target, whilst approximately five seconds of data was recorded at each distance.

Refraction data was converted to accommodation using the following equation:

\[
Accommodation(D)=-1*refraction(D)
\]

This conversion was performed by a specially designed macro (University of Reading). The macro created a graph to show accommodation response over time, and using this, we selected a vignette of data representing one second of stable data (50 data points) at each fixation distance for analysis. The macro used the selected data at the four distances to also calculate the slope of the accommodation response in relation to target demand (slope of 1.0 infers a perfect response).

### Nott dynamic retinoscopy

Nott dynamic retinoscopy was used to determine the lag or lead of accommodation. Participants were instructed to binocularly view the N5 print at 40 cm on the UC Cube. No further input was required from the participants. The retinoscope was placed alongside the target, and the reflex was observed. The least ametropic meridian was assessed. The researcher moved further back if a ‘with’ movement was seen and moved forward if an ‘against’ movement was seen until they reached the neutral point (retinal conjugate). A neutral point in front of the target indicated a lead of accommodation, and a neutral point behind the target indicated a lag of accommodation. The lead and lag of accommodation were calculated using the following equation:


\[
Lead/lag(D)=1/distance\ of\ target(m)-1/distance\ of\ retinoscope(m)
\]

This test was conducted three times for the right eye and three times for the left eye, with the average lead/lag of accommodation for each eye calculated.

### RAF Rule

The NPA was assessed using the RAF Rule push-up method. Participants viewed the N5 print on the RAF Rule and were instructed to inform the examiner when this became blurred as it was steadily pushed towards them. The test was conducted binocularly (3 times), right eye only (3 times), and left eye only (3 times). The average NPA was converted to the amplitude of accommodation for analysis using the following equation:


\[
Accommodation(D)=1/distance(m)
\]

### Statistical Analysis

Data was analysed with SPSS (version 29, Armonk, NY). A two- or three-way mixed factor ANOVA was conducted for each test, with post-hoc t-tests where required. If assumptions of sphericity were violated, the Greenhouse-Geisser statistics were quoted.

## Results

Twenty-five participants were recruited with a mean age of 20.32 ± 2.81 years (range 18–32 years). Most participants were glasses wearers (60%), 32% had no refractive error, and 8% wore contact lenses. During the period of close work, most participants chose to perform a screen-based task (48%). Paper-based tasks were performed by 20% of participants, and 32% used both paper- and screen-based tasks. For all accommodation tests, a paired t-test found no significant difference between left and right eye measurements, so the mean accommodation of the two eyes was used for all analyses.

### Plusoptix PowerRef III

**Figure 2:**
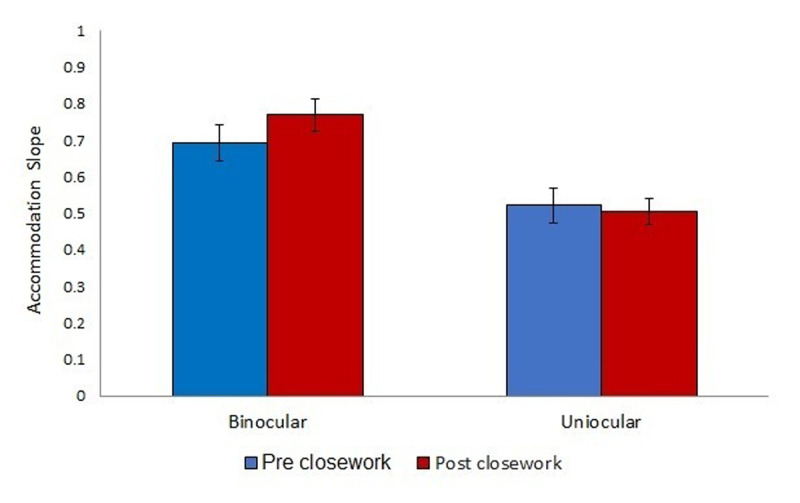
Accommodation slope across the binocular and uniocular viewing conditions pre- and post- close work, measured using the Plusoptix Power Ref III. Error bars denote standard error.

In the binocular condition, pre-close work, the mean accommodation slope was 0.69 ± 0.05. This was lower in the uniocular condition, with a mean accommodation slope of 0.52 ± 0.05. There was an improvement in accommodation slope post close-work in the binocular condition (0.77 ± 0.04; change = 0.08), but a reduction in accommodation slope in the uniocular condition (0.50 ± 0.04; change 0.02; Figure 2).

A three-factor mixed-measures ANOVA with pre/post-close work and viewing condition (binocular vs. uniocular) as the within-subject factors and type of close work activity as the between-subjects factor revealed a significant main effect of viewing condition (F (1,16) = 17.596, p < 0.001). There was no significant main effect of pre/post-close work (F (1,16) = 0.00, p > 0.05) and no significant main effect of activity type (F (2,16) = 0.462, p > 0.05). All two-way interactions were not significant (p > 0.05).

**Figure 3:**
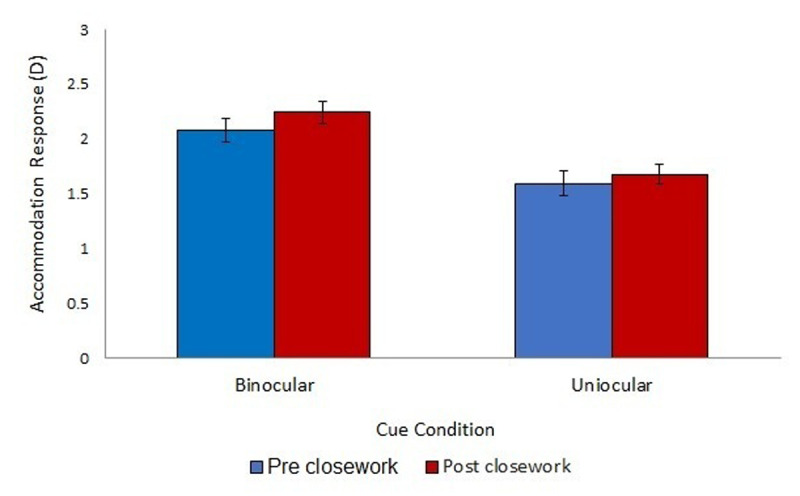
Accommodation response at 3D (33 cm) across the binocular and uniocular viewing conditions pre- and post- close work, measured using the Plusoptix PowerRef III. Error bars denote standard error.

The analysis was repeated for the accommodation response at 33 cm (demand of 3D). In the binocular condition, pre-close work, the response at 3D was 2.08 ± 0.10D (lag of 0.92D). This was lower in the uniocular condition, with a mean accommodation response of 1.59 ± 0.12D (lag of 1.41D). There was an improvement in accommodation response post-close work in the binocular condition (2.24D ± 0.11D; change = 0.16D) and a smaller improvement in the uniocular condition (1.68 ± 0.09D; change = 0.09D; Figure 3).

There was a significant main effect of viewing condition (F (1,18) = 17.891, p < 0.001). There was no significant main effect of pre/post-close work (F (1,18) = 0.815, p > 0.05) and no significant main effect of activity type (F (2,18) = 0.120, p > 0.05). All interactions were not significant (p > 0.05).

### Nott dynamic retinoscopy

**Figure 4:**
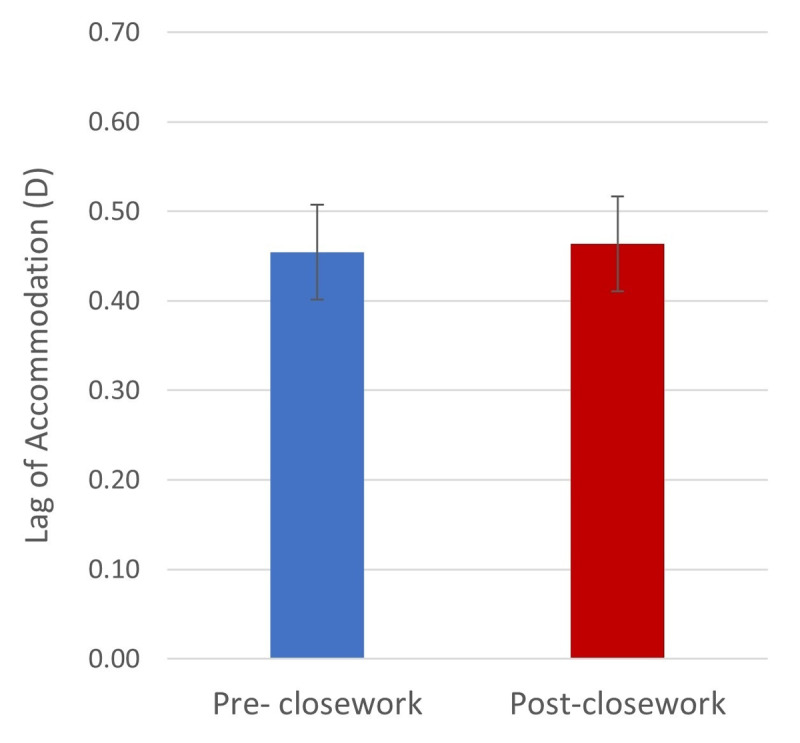
The lag of accommodation (D) measured using Nott dynamic retinoscopy pre- and post- close work. Error bars denote standard error.

Pre-close work, there was a mean 0.45 ± 0.06D lag of accommodation. This is equivalent to the point of accommodation being 10 cm behind the target placed at 40 cm. The majority of participants (92%) demonstrated a lag of accommodation. Only two participants had a lead of accommodation, measuring 0.17D and 0.35D. Post-close work, there was a mean 0.46 ± 0.06D lag of accommodation (Figure 4).

A two-way mixed ANOVA, with pre- and post-measurements as the within-subjects factor and activity type as the between-subjects factor, revealed no significant main effect of pre/post-close work (F (1,21) = 0.067, p > 0.05). Those eyes, which had a lead of accommodation pre-close work, continued to have a lead of accommodation post-close work. There was no change in the results with the removal of these two participants. There was also no significant main effect of activity type (F (2,21) = 0.163, p > 0.05) and no significant pre/post-close work*activity type interaction (F (2,21) = 1.502, p > 0.05).

### RAF Rule

**Figure 5:**
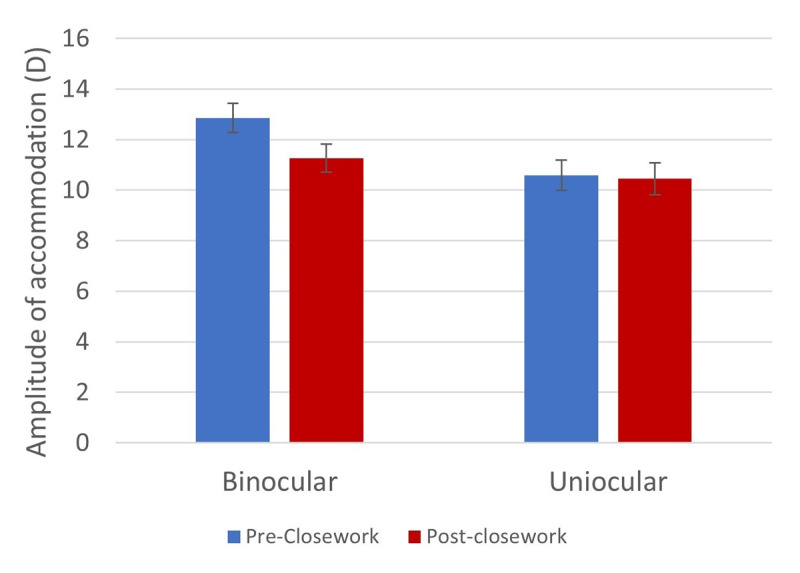
The binocular and uniocular amplitude of accommodation (D) measured using the RAF Rule pre- and post- close work. Error bars denote standard error.

The binocular amplitude of accommodation pre-close work was 12.85 ± 0.58D, which is equivalent to a NPA of 7.78 cm. A two-way mixed measures ANOVA, with pre- and post-measurements as the within-subjects factor and activity type as the between-subjects factor, revealed a significant decrease in the amplitude of accommodation after close work to 11.26 ± 0.55D (8.89 cm; F (1,22) = 8.753, p = 0.007; Figure 5). There was no significant main effect of type of activity for close work (F (2,22) = 1.089, p > 0.05) and no significant pre/post-close work*activity type interaction (F (2,21) = 0.249, p > 0.05).

When tested uniocularly, the mean amplitude of accommodation pre-close work was 10.59D ± 0.59 (9.44 cm). A two-way mixed measures ANOVA, with pre- and post-measurements as the within-subjects factor and activity type as the between-subjects factor, revealed no significant change in the amplitude of accommodation after close work (10.45 ± 0.63D; 9.57 cm; F (1,22) = 0.053, p > 0.05). There was no significant main effect of activity type (F (2,22) = 1.071, p > 0.05) and no significant pre/post-close work*activity type interaction (F (2,21) = 0.114, p > 0.05; Figure 5).

### Convergence

As only the binocular amplitude of accommodation was significantly reduced post-close work, the possibility of convergence fatigue was investigated. Convergence data from the Plusoptix was extracted and analysed. Pre-close work, in the binocular condition, the convergence slope was 0.99 ± 0.04. This reduced to 0.62 ± 0.04 in the uniocular condition.

A three-way factor ANOVA, with pre/post-close work and viewing condition (binocular vs. uniocular) as the repeated factors and type of activity as the between-subjects factor, revealed a significant main effect of cue condition (F (1,15) = 26.959, p < 0.001). There was no significant change in convergence after close work (F (1,15) = 0.30, p > 0.05) and no significant main effect of activity (F (2,15) = 0.93, p > 0.05). All interactions were not significant (p > 0.05). Statistical outcomes were the same if the convergence response at 3D was used.

## Discussion

The orthoptic clinical assessment of accommodation typically only consists of measuring the NPA using the RAF Rule, which is prone to many errors. The purpose of this study was to compare Nott dynamic retinoscopy to the commonly used RAF Rule and gold standard Plusoptix PowerRef III, to determine whether this is a more suitable test to measure accommodation.

A period of close work was used to elicit a possible change in accommodation. However, as the participants were typical young adults, as expected, the Plusoptix detected no significant change in accommodation slope or accommodation response at 33 cm after close work.

In the binocular viewing condition, the accommodation slope pre- and post- close work was 0.69 and 0.77. A slope of 1 is the ideal response, but research in a group of typical young adults using a similar lab setup detected a similar slope of 0.75 due to a lag of accommodation at near (Horwood, Toor and Riddell, 2014). Our participants also revealed a similar lag of accommodation at 33 cm, which did not significantly change following close work (pre-close work 0.92D; post-close work 0.76D). This lag of accommodation is within normal limits at 40 cm, as a lag of 0.5D and up to 1.00D is considered normal in under 40-year-olds (León et al., 2017). In the uniocular viewing condition, there was a significant reduction in accommodation slope and accommodation response at 33 cm compared to the binocular viewing conditions pre- and post-close work, due to disparity being the main cue that drives accommodation (Horwood and Riddell, 2008).

Similar to the Plusoptix, Nott dynamic retinoscopy also revealed no significant change in accommodation after a period of close work. The mean lag of accommodation pre-close work was 0.45D and the lag of accommodation post-close work was 0.46D. Nott dynamic retinoscopy was assessed at the recommended distance of 40 cm (Bhalla and Mohan, 2014). However, a limitation of the current study is that Nott dynamic retinoscopy was not also performed at 33 cm, which would have allowed for a more direct comparison with the Plusoptix measurements. The lag of accommodation increases at closer viewing distances (Seidemann and Schaeffel, 2003); therefore, testing at 33 cm may have yielded values more comparable to those obtained with the Plusoptix. Nonetheless, the results from both methods are within normal limits, and both indicated no significant change after close work.

There was a significant reduction in the binocular amplitude of accommodation measured with the RAF Rule after close work. Pre-close work, the amplitude of accommodation of 12.85D (7.78 cm) was within normal limits, as the expected amplitude of accommodation for the participant’s average age of 20.32 years using Hofstetter’s formula is 12.40D (Hofstetter, 1947). Following close work, the amplitude of accommodation decreased to 11.26D (8.89 cm), indicating accommodative fatigue on repeated testing, whilst both measures of accommodative lag did not fatigue. Although clinically the amplitude of accommodation is still within normal limits, the significant change after only 30 minutes of close work in a group of typical young adults suggests the test is prone to fatigue or errors.

However, as this was not the case, a reduction in the binocular amplitude of accommodation could have been caused by convergence fatigue. Although assessing convergence was not an objective of this study, the Plusoptix simultaneously measures accommodation, convergence and pupil size. The convergence data was extracted and analysed, revealing no significant change in convergence pre- and post- close work. There was no evidence of accommodative or convergence fatigue using the gold standard test, and the uniocular amplitude of accommodation was not affected, suggesting that the reduction in the binocular amplitude of accommodation is likely due to the subjective nature of the test.

### Clinical recommendations

The RAF Rule remains a valuable clinical test. It is an inexpensive piece of equipment that is quick and easy to use and provides a measure of the near point of convergence, as well as an estimate of the maximum accommodative ability. However, as demonstrated by the results of the current study, the test is prone to errors, likely due to its subjective nature, and primarily reflects accommodative performance at threshold levels. Nott dynamic retinoscopy provides an objective assessment of accommodative response at functional working distances, and was found in this study to be comparable to the Plusoptix PowerRef III. As it is quick to perform and uses a retinoscope that is readily available in eye clinics, Nott dynamic retinoscopy is a practical method for assessing accommodative behaviour during real-world visual tasks. Therefore, the combined use of the RAF Rule and Nott dynamic retinoscopy allows clinicians to provide a more comprehensive evaluation of patients with accommodative anomalies.

## Conclusion

The gold standard Plusoptix PowerRef III and Nott dynamic retinoscopy revealed no significant change in accommodation after a period of close work. Both objective tests were comparable, detecting a similar lag of accommodation. There was a significant reduction in the binocular amplitude of accommodation measured with the RAF Rule after close work. However, there was no significant change in the uniocular amplitude of accommodation. The Plusoptix convergence data confirmed this difference was not due to convergence fatigue and therefore is likely due to the subjective nature of the test. The changes in accommodation found with the RAF Rule confirm that the test is prone to errors. Nott dynamic retinoscopy is a more appropriate test to assess accommodation and should be used in conjunction with the RAF Rule in the assessment of accommodative anomalies.
